# A Review of Samidorphan: A Novel Opioid Antagonist

**DOI:** 10.7759/cureus.5139

**Published:** 2019-07-15

**Authors:** Amna Mohyud Din Chaudhary, Manal F Khan, Sukhbir S Dhillon, Sadiq Naveed

**Affiliations:** 1 Psychiatry, Nishtar Medical College & Hospital, Multan, PAK; 2 Psychiatry, University of Washington, Seattle, USA; 3 Psychiatry, University of Kansas Medical Center, Kansas City, USA

**Keywords:** samidorphan, opioid antagonist

## Abstract

Opioid modulators have been explored as a treatment option for psychiatric disorders, but their use has been limited due to their abuse potential. Samidorphan (SAM), a μ-opioid receptor antagonist, has gained interest due to its favorable pharmacokinetic and pharmacodynamic profile. In this review article, six electronic databases including PubMed, PsycINFO, PsycARTICLES, Scopus, Web of Science, and CINAHL were searched to find relevant human studies with a focus on different clinical aspects of SAM. SAM was used in combination with buprenorphine (BUP) to counteract the abuse potential while still maintaining effectiveness in the treatment of depression. The BUP/SAM 2 mg/2mg combination improved depression in patients with major depressive disorder (MDD). SAM's ability to mitigate the weight gain associated with olanzapine (OLZ) has also been explored. Initial studies have shown promising results in some parameters of alcohol-use disorder, while no significant benefit in the treatment of binge-eating disorder has been reported. Somnolence and gastrointestinal side effects were the most commonly observed side effects of SAM.

## Introduction and background

Opioids are widely prescribed medications to treat moderate-to-severe acute and chronic pain insufficiently controlled by other medications. Opioids exert their analgesic effect on the central and peripheral nervous system by acting on μ (MOR), κ (KOR), and δ (DOR) receptors. In total, there are four types of opioid receptors found in the body: MOR, KOR, DOR, and opioid receptor-like 1 [1]. Opioid agents are categorized into full agonists, partial agonists, mixed agonists, and antagonists based on the affinity and receptor binding at MOR. However, pharmacological agents such as BUP, tramadol, and tapentadol have a mixed mechanism [2].

Opioid prescriptions increased significantly from 2007 to 2012 by 7.3% with primary-care specialties accounting for almost 50% of the prescriptions in the United States [3]. Increased opioid prescriptions, a rising trend in opioid addiction, and the opioid-overdose epidemic have created an increased interest in the research and development of opioid agonists as well as antagonists from a public-health standpoint. Opioid-receptor antagonists, in particular, have been developed to counteract the adverse effects of opioids. These act by reversibly binding to opioid receptors with high affinity, but they have no intrinsic activity. The FDA has approved methadone, naltrexone, and BUP for the treatment of opioid-use disorder. Naloxone and naltrexone are the two most commonly used opioid-receptor antagonists. They are used primarily in the treatment of opioid overdose and in alcohol and opioid dependence, respectively [4-5]. However, the pharmacokinetic properties of these opioid antagonists, such as low bioavailability and extensive first-pass metabolism, have limited their use in clinical practice [6].

SAM (3-carboxamido-4-hydroxynaltrexone), a more recently developed, novel opioid-system modulator, primarily functions as an MOR antagonist in vivo [7]. It is structurally related to the mixed-opioid-receptor antagonist naltrexone. Compared to naltrexone, SAM has a five-fold greater affinity at MOR and much greater bioavailability when administered orally. In vitro, it has a high affinity at MOR, KOR, and DOR; and it acts as an antagonist at MOR and a partial agonist at KOR and DOR [8].

In early studies on rats, orally administered SAM (previously known as RD-0313 or ALKS33) successfully reversed the analgesia by morphine, thereby affirming its opioid-blocking ability [9]. As there is some evidence to support opioid modulation as a potential treatment target for mood disorders, SAM is currently being evaluated as a treatment option in various psychiatric diseases including MDD, schizophrenia, and antipsychotic-induced weight gain. The favorable pharmacodynamics and pharmacokinetic properties of SAM are the reason behind its role being investigated in the treatment of alcohol-use disorder and binge-eating disorder. This article provides a comprehensive overview of SAM's pharmacokinetic and pharmacodynamic properties, abuse potential, clinical efficacy for different psychiatric disorders, and possible adverse effects.

## Review

This review article provides an insight into the different clinical aspects of SAM in human studies. 

Methods

Electronic databases including PubMed, PsycINFO, PsycARTICLES, Scopus, Web of Science, and CINAHL were searched for relevant human studies with a focus on SAM in different psychiatric disorders. However, this article is a narrative review and lacks important elements of a systematic review.

Results

SAM is being tested as an adjunctive treatment in a wide range of psychiatric disorders, and has shown potential benefits in the treatment of these conditions. We have included 17 studies in this review article. Table 1 summarizes the focus of the studies, adjunct medications, study design, sample size, duration, and doses of SAM.

**Table 1:** Summary of the focus of studies, adjunct medications, study design, sample size, duration, and doses of SAM. ADTs = antidepressant treatments, BUP = buprenorphine, MDD = major depressive disorder, N/A = not available, NAL = naltrexone, OLZ = olanzapine, OXY = oxycodone, PZC = pentazocine, RCT = randomized controlled trial, SAM = samidorphan.

Studies	Focus of study	Adjunct medication	Study design	Sample size	Duration (weeks)	Dose (mg)
Shram et al., 2015 [7]	Opioid blockage	Remifentanil	RCT	25	1.3	10 and 20
Martin et al., 2019 [8]	Schizophrenia	OLZ	Phase 2, RCT	309	13	5, 10, and 20
Bidlack et al., 2018 [10]	MDD	BUP	In vitro	N/A	N/A	Not specified
Turncliff et al., 2015 [11]	Pharmacokinetics	N/A	RCT	16	1	3.7, 11, 18.6, 37.2, and 55.7
			RCT	30		10 and 20
Sun et al., 2018 [12]	Pharmacokinetics	OLZ	Phase 1, RCT, open-label	48	1	10
Sun et al., 2019 [13]	Pharmacokinetics	OLZ	Phase 1, RCT, open-label	42	5	10
Sun et al., 2019 [14]	CYP3A4 induction	OLZ and rifampin	Phase 1, open-label	24	5	10
Pathak et al., 2019 [15]	Abuse potential of SAM	OXY, PZC, and NAL	RCT	70	18	10 and 30
Pathak et al., 2019 [16]	Abuse potential of BUP/SAM	BUP	Phase 1, RCT	132	14	2, 8, and 16
Ehrich et al., 2015 [17]	MDD	BUP	RCT	13	0.4	1, 4, 8, and 6
				32	1	0.25, 0.5, 4, and 8
Fava et al., 2016 [18]	MDD	BUP	RCT	142	4	2 and 8
Fava et al., 2018 [19]	MDD	ADTs	Phase 3, RCT	385	11	0.5, 1, and 2
				407		
Thase et al., 2019 [20]	MDD	BUP	Phase 3, open-label	1,454	52	2
Silverman et al., 2018 [21]	Weight gain	OLZ	Phase 1, RCT	106	3	5
Potkin et al., 2019 [22]	Schizophrenia	OLZ	Phase 3, RCT	403	4	10
McElroy et al., 2013 [23]	Binge-eating disorder	None	RCT	62	6	10
O’Malley et al., 2018 [24]	Alcohol-use disorder	None	Phase 2, RCT	406	12	1, 2.5, and 10

Table 2 summarizes the clinical outcome of these studies.

**Table 2:** Summary of the clinical outcomes of the studies. BUP = Buprenorphine, CGI-I = Clinical Global Impression-Improvement Scale, CGI-S = Clinical Global Impression-Severity Scale, HAM-D = Hamilton Depression Rating Scale, MADRS = Montgomery–Åsberg Depression Rating Scale, MOR = μ-opioid receptor, OLZ = Olanzapine, PANSS = Positive and Negative Syndrome Scale, PGART = Patient Global Assessment of Response to Therapy, SAM = samidorphan.

Study	Clinical 0utcomes
Shram et al., 2015 [7]	Opioid antagonism detected at 15 minutes for 20 mg dose with maximum blockage at 10 mg. Initial response was achieved after 72 or 96 hours.
Martin et al., 2019 [8]	37% less weight gain in OLZ/SAM combination.
Bidlack et al., 2018 [10]	SAM decreases the efficacy of BUP on MOR.
Turncliff et al., 2015 [11]	T-max of 1 hour, half-life of 7-9 hours, steady-state reached by day 7 for SAM 10 mg.
Sun et al., 2018 [12]	Single tablet of OLZ/SAM does not affect the pharmacokinetics/bioavailability of either drug.
Sun et al., 2019 [13]	The co-administration of OLZ/SAM does not affect the pharmacokinetics of either drug.
Sun et al., 2019 [14]	Rifampin decreased the mean systemic exposure to both OLZ and SAM.
Pathak et al., 2019 [15]	The abuse potential of SAM at 10 mg and 30 mg was similar to that of naltrexone and Placebo.
Pathak et al., 2019 [16]	The BUP/SAM 2 mg/2 mg combination suggested no abuse potential.
Ehrich et al., 2015 [17]	Decrease in pupil size within four hours, improvement in HAM-D17 and MADRS scores.
Fava et al., 2016 [18]	Significant improvement on HAM-D, MADRS, and CGI-S scale in the BUP/SAM 2 mg/2 mg combination.
Fava et al., 2018 [19]	THe BUP/SAM 2mg/2mg combination showed a reduction in depression symptomatology and suicidal ideation.
Thase et al., 2019 [20]	Improved MADRS score, low rate of euphoria-related events, and no change in body weight.
Silverman et al., 2018 [21]	The OLZ/SAM combination caused significantly less weight gain over a 21-day period as compared to OLZ alone.
Potkin et al., 2019 [22]	Improvement in PANSS and CGI-I scale response at week 4, compared to OLZ or placebo.
McElroy et al., 2013 [23]	An overall significant reduction was seen in weekly binge days and frequency but without significant effect over time.
O’Malley et al., 2018 [24]	Improvement in cumulative heavy drinking days, WHO drinking-risk level, alcohol craving, and PGART.

Table 3 summarizes the adverse effects and limitations of the studies focused on SAM.

**Table 3:** Summary of adverse effects of SAM and limitations of the studies. N/A = not available, SAM = samidorphan

Studies	Adverse effects	Limitations
Shram et al., 2015 [7]	Dysgeusia, somnolence, nausea, dizziness, headache, and anorexia.	Potential bias and sequence effects.
Martin et al., 2019 [8]	Somnolence, sedation, dizziness, and constipation.	Lead-in phase, small male-only sample, and short duration.
Bidlack et al., 2018 [10]	N/A	Unclear level of signalings.
Turncliff et al., 2015 [11]	Somnolence and nausea.	Underrepresentation of female subjects.
Sun et al., 2018 [12]	Dizziness, nausea, sedation, somnolence, and tachycardia.	
Sun et al., 2019 [13]	Weight gain, dry mouth, and constipation.	Not mentioned.
Sun et al., 2019 [14]	Somnolence, dizziness, presyncope, nausea, vomiting, and hypotension.	Not mentioned.
Pathak et al., 2019 [15]	Nausea, vomiting, decreased appetite, somnolence, headache, altered mood, pruritus, and dizziness.	Not mentioned.
Pathak et al., 2019 [16]	Nausea and vomiting.	Lack of use of multiple doses.
Ehrich et al., 2015 [17]	Nausea, vomiting, and dizziness.	Small sample size.
Fava et al., 2016 [18]	Nausea, vomiting, dizziness, and headaches.	Short duration and small sample size.
Fava et al., 2018 [19]	Nausea, constipation, dizziness, vomiting, somnolence, fatigue, and sedation.	
Thase et al., 2019 [20]	Nausea, headache, constipation, dizziness, and somnolence.	Short duration, small sample size, and no long-term follow-up.
Silverman et al., 2018 [21]	Orthostatic hypotension, somnolence, and nausea.	Not mentioned.
Potkin et al., 2019 [22]	Weight gain, somnolence, dry mouth, anxiety, and headache.	
McElroy et al., 2013 [23]	GI distress, dizziness, headache, insomnia, dry mouth, sedation, hallucinations, and palpitations.	Not mentioned.
O’Malley et al., 2018 [24]	Nausea.	Male volunteers, small sample size, the single dose level of SAM.

Pharmacokinetics and pharmacodynamics

An in vitro study by Bidlack et al. (2018) investigated the effect of SAM and BUP on opioid receptors. This study showed that both SAM and BUP bind with MOR and KOR receptors with high affinity. SAM was found to be an antagonist at MOR and a partial agonist at KOR. When the BUP/SAM combination was used, SAM decreased BUP's effect on MOR without having an impact on BUP's effect on KOR [10]. In human studies, SAM was rapidly absorbed and achieved its maximum serum concentration (Cmax) in about one hour; the area under the curve (AUC) increased in a dose-dependent manner. Its elimination occured largely through hepatic CYP3A4-mediated metabolism as well as renal excretion. The plasma levels of SAM decreased in a mono-exponential manner. The half-life of SAM was found to be about 7-9 hours. A steady state was approached by day 6 after multiple doses, and achieved by day 7 for SAM 10 mg [11]. A study by Sun et al. (2018) showed that the OLZ/BUP (ALKS 3831) combination had no effect on the pharmacokinetic profile (including T-max and T ½) of either drug [12-13]. Sun et al. (2019) evaluated the metabolism and elimination of the OLZ/SAM combination when co-administered with rifampin. Although the median time to reach maximum concentration remained the same, shorter half-life was observed thanks to increased mean clearance due to rifampin. The AUC for SAM was lower due to rifampin’s effect on CYP3A4 [14].

Abuse potential

Pathak et al. (2019) studied the abuse potential of SAM at 10 mg and 30 mg. At both doses, the visual analog scores (VAS) for at-the-moment and overall-drug-liking were similar to those of naltrexone and placebo. However, the take-drug-again scores as well as the minimum-effect alertness/drowsiness VAS scores were greater than those of placebo but similar to those of naltrexone. Pupillometry also affirmed the lack of abuse potential of SAM [15]. Another study by Pathak et al. (2019) investigated the abuse potential of the BUP/SAM combination. No abuse potential was seen at 2 mg/2 mg combination across the drug-liking VAS, overall-drug-liking, and take-drug-again scales. Even at higher doses of 8mg and 16mg, the abuse potential was lower compared to BUP alone, suggesting that SAM mitigated the abuse potential of BUP [16-17].

Depression

There is always a need to develop new drugs for the treatment of depression resistant to currently available medications. SAM has therefore been investigated for its potential role in the treatment of MDD. Fava et al. (2016) investigated the BUP/SAM combination in the treatment of MDD in a randomized, double-blind, placebo-controlled, and sequential parallel study. This four-week-long study investigated the effects of SAM at doses of 2 mg and 8 mg. A remarkable improvement was seen in depressive symptoms at the BUP/SAM 2 mg/2 mg combination whereas the improvement was little for the BUP/SAM 8 mg/8 mg combination [18]. FORWARD-4 and FORWARD-5 studies by Fava, et al. in 2018 investigated the efficacy of the BUP/SAM combination in MDD at doses of 0.5, 1, and 2 mg. These studies compared the therapeutic effect of the BUP/SAM combination with that of standard antidepressant drugs. The pooled analysis confirmed the efficacy of the BUP/SAM 2 mg/2 mg combination over placebo. There was a decrease in suicidal ideation in patients receiving the BUP/SAM combination compared to those on other ADTs [19]. In 2019, Thase et al. conducted a Phase-3, open-label study using the BUP/SAM combination for a duration of 52 weeks in a large sample size of 1,454 patients. The mean MADRS score was found to decrease from a baseline of 22.9 (±9.7) to 9.8 (±8.8). There was also no evidence of withdrawal on abrupt discontinuation or increase in suicidal ideation or behavior [20].

Schizophrenia

A number of studies have been conducted using SAM as an adjunct in schizophrenia. In an early Phase-1 study by Silverman et al. (2017), the OLZ/SAM combination caused significantly less weight gain compared to OLZ alone. A similar trend was observed in metabolic tests including triglycerides, cholesterol, and the glucose-to-insulin ratio. SAM clearly reduced the unwanted adverse effects seen with the use of OLZ alone [21]. Later, a 13-week-long, phase-2 study by Martin et al. showed similar results: patients experiencing 37% less weight gain when on the OLZ/SAM combination, compared with OLZ and placebo [8]. In 2019, Potkin at al. used SAM 10 mg as an adjunct with OLZ for four weeks. The results were promising, with improvement in psychotic symptoms with ALKS 3831 (a combination of a flexible dose of olanzapine with a fixed 10 mg dose of SAM) [22].

Binge-eating-disorder

A study of SAM by McElroy et al. (2013) showed no significant reduction in weekly binge frequency or weekly binge days, body weight, body-mass index, waist circumference, or other measures of disease pathology and severity [23].

Alcohol dependence

O’Malley et al. (2018) studied the effect of different doses of SAM on alcohol dependence. Although there was no significant improvement in the primary outcome measure, improvement was noted for cumulative heavy-drinking days, alcohol craving, and PGART. There was also improvement in WHO drinking-risk level over the 12-week-study period compared with placebo. SAM 10 mg was found to have the maximum clinical effect [24].

Opioid antagonism

SAM blocks the opioid-agonist effect of remifentanil. The effect was seen occuring at around 15 minutes with SAM 10 mg and persisted for up to 24 hours. The elimination half-life for SAM was around seven hours, which is greater than that for naltrexone (four hours). Remifentanil took up to 72-96 hours to exert full response after the co-administration of SAM and remifentanil. The plasma concentration of SAM became undetectable after 72-96 hours [7].

Adverse effects

The adverse effects of SAM were minimal. Euphoria was only seen in 1.2% of the patients. There were no significant changes in metabolic parameters or body weight [19]. Compared to other opioid antagonists, SAM was generally well-tolerated and somnolence was the most commonly reported adverse effect in different studies. Other reported adverse effects included nausea, constipation, and decreased appetite [11]. Other less commonly reported adverse effects included dry mouth, anxiety, headache, and orthostatic hypotension [20-21].

Discussion

As discussed above, SAM is a novel opioid-system modulator that acts as an opioid antagonist at MOR in vivo. Although chemically derived from naltrexone [25], SAM has a greater binding affinity for MOR compared to naltrexone. It also has a longer elimination half-life compared to naltrexone when orally administered [7]. These properties make SAM more effective as an MOR antagonist.

In the practice of psychiatry, there is always a need for new treatment options. The existing psychotropic drugs are known to widely cause adverse side effects, which can lead to compromising medication adherence. They can also cause an incomplete resolution of symptoms, which may necessitate many different medication trials and adjunct treatments. Our review establishes that SAM has the most potential as an adjunct medication in combination with BUP for the treatment of MDD [18-20]. We also demonstrate that SAM can help in the attenuation of the weight gain caused by OLZ in the treatment of schizophrenia [8, 21]. However, the evidence we have enumerated in the review should be considered in the context of the lack of approval from the FDA for the use of the BUP/SAM combination in the treatment of depression, citing abuse potential as a concern [26].

About 40% of the patients experiencing MDD fail to achieve full remission [18]. The novel mechanisms for antidepressant response, such as opioid, N-methyl-d-aspartate, or gamma-aminobutyric acid receptor modulation excite psychiatrists as it represents the potential for new strategies for the treatment of unipolar depression. The SAM/BUP combination used as an adjunct to anti-depressant medications in the treatment of MDD has shown an improvement in depressive symptomatology [18]. The BUP/SAM 2 mg/2 mg combination has been found to be the most effective. SAM considerably mitigates the abuse potential of BUP [16]. Although OLZ is regarded as one of the most efficacious antipsychotic medications, the metabolic side effects of the medication negatively impact its use. The OLZ/SAM combination has been developed to attenuate the weight gain caused by OLZ. Patients who have used the OLZ/SAM combination have had less weight gain than those who have used OLZ without SAM [8, 21]. Further, the addition of SAM does not impact the pharmacokinetics and efficacy of OLZ [12]. 

SAM has been studied in the treatment of other psychiatric disorders including binge-eating disorder, alcohol abuse. Its abuse potential has also been investigated. Unfortunately, in binge-eating disorder, SAM has not shown improvement in most parameters. For alcohol-use disorder, the primary-outcome measure did not show significant improvement; however, improvement has been observed in other parameters such as cumulative heavy-drinking days, change in WHO drinking-risk level, alcohol-craving, and PGART [24]. SAM has been established as having no abuse potential in the doses recommended in the treatment of psychiatric disorders. 

Somnolence is the most commonly reported side effect of SAM. The limitations of most studies include small sample size, short duration, and limited heterogeneity amongst patients/subjects. From being used in the treatment of psychiatric disorders to mitigating side effects of psychotropic medications, SAM has shown potential for a wide variety of use.

## Conclusions

This article reviews the different clinical aspects of SAM in human studies. We have tried to demonstrate that the use of the SAM/BUP combination in the treatment of several psychiatric disorders has a greater efficacy with much less abuse potential. It was also helpful in mitigating antipsychotic-induced weight gain associated with OLZ, but without compromising on the efficacy or pharmacokinetics of OLZ. There is also possible evidence of improvement in alcohol-use disorder in certain outcome measures. However, SAM has failed to provide benefits in patients with binge-eating disorder. Somnolence and gastrointestinal side effects are the most commonly observed side effects of SAM. However, the review should be considered in the context of the lack of approval from the FDA for the BUP/SAM combination for the treatment of depression, citing abuse potential as a concern.
